# Anxiety, perceived stress and coping strategies in nursing students: a cross-sectional, correlational, descriptive study

**DOI:** 10.1186/s12909-020-02294-z

**Published:** 2020-10-19

**Authors:** María Dolores Onieva-Zafra, Juan José Fernández-Muñoz, Elia Fernández-Martínez, Francisco José García-Sánchez, Ana Abreu-Sánchez, María Laura Parra-Fernández

**Affiliations:** 1 Department of Nursing, Physiotherapy and Occupational Therapy, Nursing Faculty of Ciudad Real, Universidad de Castilla-La-Mancha, Ciudad Real, Spain; 2 Department of Psychology, Universidad Rey Juan Carlos I, Alcorcón, Madrid, Spain; 3 Department of Nursing, University of Huelva, Huelva, Spain

**Keywords:** Anxiety, Coping behavior, Nursing students, Perceived stress

## Abstract

**Background:**

For many nursing students, clinical training represents a stressful experience. The levels of stress and anxiety may vary during students’ educational training, depending on their ability to adopt behavioral strategies for coping with stress, and other factors. This study aimed to investigate the relationship between anxiety, perceived stress, and the coping strategies used by nursing students during their clinical training.

**Methods:**

A cross-sectional correlational descriptive study. The sample consisted of 190 nursing students enrolled in the Nursing Faculty of Ciudad Real University in Spain. Participants provided data on background characteristics and completed the following instruments: the Perceived Stress Scale; the State-Trait Anxiety Inventory and the Coping Behavior Inventory. Relationships between scores were examined using Spearman’s rho.

**Results:**

The mean age of participants was 20.71 ± 3.89 years (range 18–46 years). Approximately half of the students (47.92%) indicated a moderate level of stress with a mean Perceived Stress Scale score of 22.78 (±8.54). Senior nursing students perceived higher levels of stress than novice students. The results showed a significant correlation for perceived stress and state anxiety (*r* = 0.463, *p* < .000) and also for trait anxiety (*r* = 0.718, *p* < .000). There was also a significant relationship between the total amount of perceived stress and the following domains of the coping behavior inventory: problem solving (*r* = −.452, *p* < .01), self-criticism (*r* = .408 *p* < .01), wishful thinking (*r* = .459, *p* < .01), social support(*r* = −.220, *p* < .01), cognitive restructuring (*r* = −.375, *p* < .01), and social withdrawal (*r* = .388, *p* < .01). In the current study, the coping strategy most frequently used by students was problem-solving, followed by social support and cognitive restructuring.

**Conclusions:**

Nursing students in our study presented a moderate level of stress, in addition there was a significant correlation with anxiety. Nursing teachers and clinical preceptors/mentors should be encouraged to develop programs to help prepare nursing students to cope with the challenges they are about to face during their clinical placements.

## Background

Research on stress levels among health professionals is an issue of current interest that merits concern [1]. This is not only due to the many causes of intrinsic stress referred by healthcare professionals, but also because it is worth considering the negative and chronic effects of stress over time [2].

In nursing, the subject of stress has received much attention in the literature, and continues to be the topic of many studies [3, 4]. The practical training of a nurse’s education has been reported to be much more stressful than academic training. Also, the perceived lack of knowledge and skills are considered to be one of the common stressors for many students [5]. Furthermore, the first experience in clinical practice includes stressors such as fear of making mistakes, having to handle emergency situations, irregularities in clinical practice and visiting specialized units [6].

Generally, nursing students do not have the same responsibility in the individual care of patients in clinical practice as registered nurses, however they are exposed to some of the same stressors. Examples of the same include the relationships with other professionals, the notorious ranking that exists in hospitals, difficult situations regarding the treatment of patients and dealing with family members and the way they experience the death of the patients they care for [7]. Furthermore, nursing students coexist with other stressors that are typical considering their role as students, such as those related with their academic program and their role as nursing students [8]. This is because, as opposed to other degree programs, nursing students are in touch with the job market which requires a certain responsibility in the wellbeing of their patients, distancing them, at times from the student campus life and especially, from normal social activities enjoyed by their peers.

Low or moderate levels of stress may enhance students’ motivation, leading to greater perseverance when studying and achieving future goals [9]. Conversely, high levels of stress can have a negative influence on students, leading to depression and despair, and therefore affecting students’ health and academic level [10]. Stress is unavoidable and, in most cases, it is difficult to overcome, however, a good coping strategy may help students to improve their academic results [11].

Coping mechanisms are essential when trying to deal with the stress and anxiety that nursing students face on a daily basis. Longitudinal studies have shown that stress levels in nursing students may increase or decrease during their educational training depending on coping behavior strategies. However, as noted by Jimenez et al. [12], these differences regarding stress levels over the course of professional training should be considered with caution, as different programs exist in each country. Whereas some studies identify the first or second year as being the most stressful for students [13], for others, the third year is the most stressful due to the clinical duties [14, 15]. However, other studies show that stress levels increase according to the training or the academic year [16, 17] or decrease as the student becomes more trained [18]. Besides these differences, coping strategies vary according to the characteristics of the individual and the context where the stressors are found. The use of problem solving strategies has been identified as one of the best ways to cope with stress. Conversely, emotional based coping strategies appear to be the least effective strategy [19]. Therefore, the main aim of this study was to investigate the relationship between anxiety, perceived stress and coping strategies used by nursing students during their clinical training. Moreover, we performed a comparative analysis between perceived stress and coping strategies by gender, course and clinical placement, based on the available literature.

## Methods

### Study design, setting and participants

A cross-sectional, descriptive, correlational design was used. Graduate-level students were recruited from the Ciudad Real Nursing Faculty of Castilla-La Mancha University during the 2017/18 academic year. Currently, in Spain the nursing degree lasts 4 years with a total of 240 credits, under the European Credits Transfer System (ETCS). At the University of Castilla-La-Mancha (UCLM), during the first academic year, nursing students only have academic subjects (theoretical-practical taught in the classroom setting). These classes take place at the Faculty, and, at this point students have no contact with hospital settings. After the first year, once the basic core subjects are taught, the students begin their clinical placements. These begin in the first semester of the second year and are completed in the fourth year, with subjects that are entirely care-based and which take place at the hospital (Table 6 in Appendix). During these 3 years, students alternate clinical placements with academic subjects.

A prior sample size calculation was not performed for this study, rather the sample size was based on the entire population of students, as described previously. Clinical placements are an essential part of the acquisition of competencies and skills for nursing students, constituting an important element of their education.

The eligibility criteria for this study were as follows: all participants were students enrolled in an academic year of the nursing degree course taught at the university (except first year students). Participants must have been present in the classroom when the researcher visited to collect data. Furthermore, informed consent was required for participation. The population of nursing students studying at the university was 340 students, of these, 115 were excluded because they did not fulfill the inclusion criteria; thirty-three did not participate in the research and, finally, 192 students agreed to participate and complete the self-administered questionnaire. The response rate was 85.33%.

### Data collection instruments

Data were collected from the students in the study group using a 20- min online self-report questionnaire comprising the following information:

- Demographic characteristics: The demographic questionnaire was constructed by the researchers and was based on the recent literature. This included items such as age, gender, relationship status, academic year of the nursing degree etc.
- Perceived stress scale (PSS). The PSS-14 was designed for measuring the degree to which daily life situations are evaluated as stressors. The PSS-14 consists of multiple choice questions measuring stressful experiences and responses to stress over the previous 4 weeks. The European Spanish version PSS (14-item) has demonstrated adequate reliability (internal consistency, alpha = .81) [20]. Our study demonstrated an alpha =. 87. The range for total scores on the PSS-14 was from 0 to 56. Stress scores below the 25th percentile (0 to 17) were interpreted as low stress, scores between the 25th and 75th percentile (18 to 28.5) were interpreted as moderate stress and scores above the 75th percentile (28.6 to 56) were interpreted as high stress. On the Stress Survey, each item’s mean stress rating was calculated. An item with an average of 5 was interpreted as causing “severe stress”, 4 as causing “a lot of stress”, 3 represented “moderate stress”, 2 was “a little stress”, and 1 equaled “no stress”.
- The State-Trait Anxiety Inventory (STAI): Internal consistency coefficients for the Spanish version scale range from .86 to .95 [21]. In our sample we obtained an alpha of 0.91 for STAI State and an Alpha of 0.86 for STAI trait. The STAI has 40 items with 20 items allocated to each of the subscales, based on a 4-point Likert format (score from 0 to 3). The range of scores for each subscale is 0–60, in which higher scores indicate greater anxiety. The State Anxiety Scale (S-Anxiety) evaluates the current state of anxiety, whereas the Trait Anxiety Scale (T-Anxiety) evaluates relatively stable aspects of “anxiety proneness,” including general states of calmness, confidence, and security [21].
- The Coping Strategy Inventory (CSI) is a self-report questionnaire designed to assess coping thoughts and behaviors in response to a specific stressor [22]. We used the existing Spanish version [22]. The internal consistency coefficients were between .63 and .89 [23] and between .64 and .85 in our sample for each primary subscale. The CSI has 40 items, each item on the CSI may be scored using a 5-point Likert format (scores from 0 to 4), with the total score ranging from 0 to 160. This questionnaire contains eight primary subscales. These are: problem solving, self-criticism, expression of emotions, wishful thinking, social support, cognitive restructuring, problem avoidance, and social withdrawal.

### Statistical data analyses

#### Descriptive analysis and reliability

Data analyses were carried out using the Statistical Software Package for the Social Sciences (SPSS) version 23.0. Data were examined by calculating the means, standard deviation (±SD), absolute and relative frequencies and percentages, in order to generate a descriptive statistical analysis. In order to measure the internal consistency and homogeneity of the three questionnaires, the Cronbach’s alpha test was performed, accepting a coefficient ≥ 0.70 as an ideal value. The individual analysis of each item was carried out using the homogeneity index which was assessed using the Spearman correlation coefficient. Each item that obtained a coefficient > 0.30 was considered useful for evaluating the attribute. Additionally, no items failed to fulfil this condition.

#### Correlations and regression analysis

After checking the non-normal distribution of the total scores of the scales in our sample using the Kolmogorv-Smirnof test, the relevant non parametric tests were used for the comparison of means between groups according to gender, course, clinical placement and previous training in health sciences.

Correlations between the scores of the different scales used were assessed using the Spearman’s rho correlation coefficient. The accepted confidence interval was 95% and the significance level for all analyses was set at *p* < .05; moreover, with the significant correlation between the perceived stress scale and the STAI and CSI a hierarchical regression model was applied to assess the independent variables that contributed significantly to the variance in the score on the PSS. These independent variables were entered into the regression model in five steps. Changes in R^2^ were reported after each step of the regression model to further determine the association of the additional variables. The significance criterion of the critical F value for entry into the regression equation was set at *p* < .05, and was considered significant in all tests.

## Results

### Descriptive results for the perceived stress scale, and dimensions of the anxiety and coping scale

The mean age of participants was 20.71 ± 3.89 years (range 18–46 years). Most students were female (86.5%) and 17.7% of the students had previous training in health sciences. Up to 52.1% of students were undergoing their first clinical placement in the second year Table 1.

**Table 1:** Means, standard deviation and skewness and kurtosis of the participants’ scores for perceived stress (PSS), anxiety (STAI) and dimensions of coping (CSI)

Constructs	Dimensions	M	SD	Min	Max	Skewness	Kurtosis
Stress	Perceived stress	22.78	8.53	5	47	.303	.155
Anxiety	Anxiety state	17.63	9.01	3	54	.894	.813
	Anxiety trait	20.13	8.73	4	46	.541	−.126
Coping	Problem solving	15.08	3.43	4	20	−.450	−.072
	Self-criticism	6.54	4.56	0	20	.484	−.152
	Expression of emotion	9.88	4.37	0	20	−.127	−.406
	Wishful thinking	9.45	5.15	0	20	.248	−.863
	Social support	13.9	4.61	0	20	−.708	.067
	Cognitive restructuring	12.51	3.78	2	20	−.212	−.188
	Problem avoidance	7.36	1.62	3	13	−.018	.560
	Social Withdrawal	6.28	3.70	0	19	.495	.012

*N* = 192; Skewness Standard Error = .175; kurtosis Standard Error = .349

The mean PSS score was 22.78 (±8.54), indicating a moderate level of stress. Furthermore, the stress scores ranged from 5 to 47 out of a possible 56. In our study, most participants (47.92%) indicated a moderate level of stress. The Anxiety state score was 17.64 (±9.01) classified as ‘no problem’ with a minimum score of 3 and maximum of 54 and Anxiety trait of 20.13 (±8.74) classified as ‘mild anxiety’ with a minimum score of 4 and maximum score of 46.

### Comparative analysis between PSS, STAI and clinical placement

Regarding the type of clinical placements, no significant differences were found when comparing the mean perceived stress (*p* = .352) using the ANOVA. However, significant differences were identified in relation to anxiety state (*p* = .002). When comparing clinical placements two by two, statistically significant differences were identified between Primary Care and Special Services (15.9 ± 8.75 vs 23.77 ± 11.16, *p* = .006), Geriatrics and Special Services (16.18 ± 7.53 vs 23.77 ± 11.16, *p* = .004) and Internal Medicine and Special Services (16.14 ± 7.75 vs 23.77 ± 11.16, *p* = .001). Up to 100% of the students who displayed severe anxiety in the state STAI were in specialized services Table 2.

**Table 2:** Comparison of the level of stress and anxiety classified by type of clinical placement

	STAI STATE (Mean ± SD)	PERCEIVED STRESS (Mean ± SD)
Geriatrics	16.18 ± 7.53	24.35 ± 8.65
Mental health	19.40 ± 10.72	25.40 ± 6.87
Primary care	15.9 ± 8.75	22.33 ± 7.30
Internal medicine	16.14 ± 7.75	21.13 ± 9.08
Specialized services	23.77 ± 11.16	23.64 ± 9.00
Mother-child health	18.27 ± 7.92	24.09 ± 7.03
TOTAL	17.64 ± 9.01	22.79 ± 8.54

### Relationships between gender, academic year and dimension of CSI

The CSI displays significant differences between gender for the dimensions Expression of emotion, Social support and Problem avoidance, as, in all cases, the mean of these scores was higher among female students. However, for the total score, despite the fact that the mean score was higher in women (23.22 ± 8.55) than in men (20 ± 8.04), significant differences were not found regarding gender (*p* = .069) for the total score. Regarding the students’ academic year, the dimensions that were found to be statistically significant were wishful thinking and social withdrawal. The total mean score of the test for students in their second year was 21.30 ± 8.65 and the total mean score for students in their third year was 24.40 ± 8.16, the statistical analysis was significant (*p* = 0.009) (Table 3).

**Table 3:** Bivariate analysis of the mean score for the dimensions of the CSI compared by academic year and gender

	Gender	(M, SD)	U	*p* value	Year	(M-SD)	U	p value
Problem solving	Female	15.22 ± 3.42	1828.50	.208	2nd	15.33 ± 3.49	4192.50	.287
	Male	14.23 ± 3.50			3rd	14.83 ± 3.39		
Self-criticism	Female	6.59 ± 4.61	2051.00	.684	2nd	5.98 ± 4.35	3952–50	.091
	Male	6.27 ± 4.30			3rd	7.16 ± 4.74		
Expression of emotion	Female	10.23 ± 4.34	1432.01	.006**	2nd	10.14 ± 4.28	4390.00	.584
	Male	7.61 ± 3.98			3rd	9.60 ± 4.48		
Wishful thinking	Female	9.59 ± 5.25	1911.00	.348	2nd	8.62 ± 5.28	3610.50	.010*
	Male	8.58 ± 4.51			3rd	10.36 ± 4.90		
Social support	Female	14.14 ± 4.65	1594.00	.032*	2nd	14.01 ± 4.65	4465.00	.725
	Male	12.38 ± 4.15			3rd	13.78 ± 4.60		
Cognitive restructuring	Female	12.68 ± 3.87	1703.50	.083	2nd	12.51 ± 3.83	4543.50	.883
	Male	11.42 ± 3.07			3rd	12.51 ± 3.76		
Problem avoidance	Female	7.47 ± 1.56	1647.00	.048*	2nd	7.47 ± 1.71	4270.00	.382
	Male	6.69 ± 1.89			3rd	7.25 ± 1.52		
Social withdrawal	Female	6.27 ± 3.66	2135.50	.932	2nd	5.50 ± 3.30	3509.00	.004*
	Male	6.34 ± 4.03			3rd	7.13 ± 3.94		

*p < .05; ** *p* < .01; U = U Mann Whitney

### Correlation and hierarchical regression analysis

A Spearman’s rho correlation was used to investigate the relationship between total perceived stress and anxiety (state and trait) and the PSS score with the total score on the CSI and all subscales. The results displayed a significant correlation for the total on the PSS and the state STAI (*r* = .463, *p* < .01) and for the total PSS and the trait STAI (*r* = .718, *p* < .01). Regarding the perceived stress and the coping strategies, the results revealed a significant relationship between the total perceived stress and the following domains of the CSI: problem solving (*r* = −.452, *p* < .01), self-criticism(*r* = .408 *p* < .01), wishful thinking (*r* = .459, *p* < .01), social support (*r* = −.220, *p* < .01), cognitive restructuring (*r* = −.375, *p* < .01), and social withdrawal (*r* = .388, *p* < .01). (Table 4).

**Table 4:** Spearman’s correlation coefficient between perceived stress, STAI state and trait and dimension of CSI

	1	2	3	4	5	6	7	8	9	10
1. Perceived stress										
2. Anxiety state	.463**									
3. Anxiety trait	.718^**^	.264^**^								
4. Problem solving	−.452^**^	.050	−.180^*^							
5. Self criticism	.408^**^	.144^*^	.316^**^	−.281^**^						
6. Expression of emotion	−.086	.051	−.063	.269^**^	.059					
7. Wishful thinking	.459^**^	.078	.313^**^	−.142	.534^**^	.196^**^				
8. Social support	−.220^**^	.031	−.088	.439^**^	−.042	.591^**^	.085			
9. Cognitive restructuring	−.375^**^	.003	−.145^*^	.573^**^	−.214^**^	.298^**^	−.071	.471^**^		
10. Problem avoidance	.105	.423^**^	.461^**^	.053	.168^*^	.106	.120	.004	−.053	
11. Social Withdrawal	.388^**^	.138	.289^**^	−.266^**^	.396^**^	−.104	.424^**^	−.275^**^	−.115	.091

* *p* < .05. ** *p* < .01

Table 5 shows the hierarchical regression analysis developed in this study.

**Table 5:** Summary of Stepwise Regression Analyses to determine predictors of Perceived stress

Independent Variables	B	SE B	β	t
Step 1				
Anxiety trait	.709	.049	.726	14.56**
Step 2				
Anxiety trait	.609	.049	.624	12.32**
Wishful thinking	.440	.084	.266	5.26**
Step 3				
Anxiety trait	.550	.049	.564	11.28**
Wishful thinking	.436	.080	.263	5.47**
Cognitive restructuring	−.475	.105	−.211	−4.53**
Step 4				
Anxiety trait	.474	.053	.486	8.98**
Wishful thinking	.445	.078	.269	5.72**
Cognitive restructuring	−.485	.102	−.215	−4.75**
Anxiety state	.153	.046	.162	3.30*
Step 5				
Anxiety trait	.439	.054	.450	8.15**
Wishful thinking	.444	.077	.268	5.79**
Cognitive restructuring	−.300	.124	−.133	−2.41*
Anxiety state	.161	.046	.170	3.52**
Problem solving	−.364	.142	−.147	−2.56*

*R*^2^ = .528 for step 1; *R*^2^ = .588 for step 2; *R*^2^ = .628 for step 3; *R*^2^ = .649 for step 4; *R*^2^ = .661 for step 5. * *p* < .05; **p < .01

For the first step, the adjustment index of the model was significant F (1, 191) 212.186, *p* < .01 and the anxiety trait variable was a significant predictor of the perceived stress scale (β = .726, *t* = 14.56, *p* < .01).

The significant variables included in the second model were: anxiety trait (β = .624, *t* = 12.32, *p* < .01) and wishful thinking (β = .266, *t* = 5.26, *p* < .01), the adjustment was F (2, 191) 134.82, *p* < .01.

For the third model, the significant variables were: anxiety trait (β = .564, *t* = 11.28, *p* < .01), wishful thinking (β = .263, *t* = 5.47, *p* < .01), cognitive restructuring (β = −.211, *t* = − 4.53, *p* < .01) and the adjustment was: F (3, 191), 106.01 *p* < .01.

For the fourth model anxiety trait (β = .486, *t* = 8.98, *p* < .01), wishful thinking (β = .269, t = 5.72, *p* < .01), cognitive restructuring (β = −.215, t = − 4.75, *p* < .01), and anxiety state (β = .162, t = 3.30, *p* < .05) were significant variables, and the adjustment was: F (4, 191) 86.44, *p* < .01.

For step 5: anxiety trait (β = .450, t = 8.15, *p* < .01), wishful thinking (β = .268, t = 5.79, *p* < .01), cognitive restructuring (β = −.133, t = − 2.41, *p* < .05), anxiety state (β = .170, t = 3.52, *p* < .01), and problem solving (β = −.147, t = − 2.56, *p* < .05) were significant variables, furthermore the adjustment was: F (5, 191) 72.53, *p* < .01. The fifth model explained 66.1% of the variance in perceived stress, representing the model with the best fit.

## Discussion

This study was conducted to assess nursing students’ perceived stress levels and their association with anxiety, as well as the coping behaviors used to reduce the effect of stress during clinical training.

Overall, 47.92% of the students experienced a moderate level of perceived stress and only 25% perceived a high degree of stress. Furthermore, the correlation between perceived stress and anxiety was significant in the present study, i.e. students with high scores of perceived stress had higher anxiety scores.

The prevalence of stress among nursing students found in the literature is variable, which could be due to the different academic programs available worldwide and the use of different scales for measuring the same [24, 25]. However, stress levels may also become affected because of different perceptions regarding stress across cultures and among different individuals. Lazarus and Folkman defined stress as “a particular relationship between the person and the environment that is appraised by the person as taxing or exceeding his or her resources and endangering his or her well-being” [26]. Besides these academic and individual differences, according to this definition of stress, it is important to include the effects of the environment, and more concretely, in our case, the different clinical placements the students visited throughout their training, on the levels of stress or anxiety presented by the student. Regarding other specialized services such as intensive care or emergency care, our study suggested that the students had higher levels of anxiety and stress during these clinical placement units.

Regarding the year of studies, our findings support previous studies where the students with the most experience displayed higher levels of anxiety, whereas the most inexperienced showed lower levels of stress and anxiety [16, 17]. This may be because students feel that their teachers and other nurses expect more from them as they are more experienced and therefore more knowledgeable students, thus increasing their stress levels. However, this interpretation should be linked to the previously cited findings, i.e., considering the clinical placements performed by the students. For example, clinical placements in more specialized services are usually completed during the later years, whereas during the first years of study, training takes place in more general services requiring more basic competencies for care and patient responsibility. Therefore, students who have more extensive training, but who are also required to have a greater level of competencies and skills during patient care are those that are exposed to a greater level of anxiety and stress. However, as suggested by Jimenez et al. [12], it is important to exercise caution in these interpretations as, in this sense, the different training programs, despite being based on the ECTS system, should not be centered only on the number of credits to be completed each year, rather, they should insist on coordinating and developing parallel competencies over time, as differences in training programs exist even within the same country.

In the current study, the coping strategies most frequently used by students were problem-solving, followed by social support and cognitive restructuring. According to Folkman and Lazarus, problem solving is one of the more effective ways to deal with stress as it focuses on behaviors in order to manage or alter the problem [26]. Problem solving has been found to be the most utilized coping strategy in different studies with nursing students [27–29], despite the fact that these studies have used a measurement scale for facing stress that is different to the one used in this study. In terms of the relationship between perceived stress and coping strategies, our findings indicate that among these three domains (problem solving, cognitive restructuring and social support) an inverse correlation exists, indicating that people who suffer less stress, will use these strategies more often. Similarly, the positive correlation with the following domains shows how people with greater stress have more anxiety trait and state and use strategies such as wishful thinking, self-criticism, social withdrawal and problem avoidance. The results of this study showed that the greatest predictor of perceived stress was anxiety trait. As for the domains or strategies used to cope with stress, in our study, the use of certain strategies, such as problem solving and cognitive restructuring, were considered to be predictors of less stress, whereas the use of wishful thinking appeared as a predictive factor of greater stress. Former studies using other coping tools found a positive relationship in terms of protection regarding the student’s mental health, in those students who used an optimistic strategy. In this sense, possibly, our study sample did not understand, culturally speaking, what wishful thinking meant or they did not know of any strategies truly framed in this state of optimism or illusion. This could be a protective factor in terms of mental health, however this may hamper optimal results in terms of reducing the stress they suffer [30].

In a qualitative study by Lopez V et al., nursing students of a University in Singapore reported that talking about their negative emotions with their peers and positive reframing of their negative circumstances were the most used strategies when facing perceived stress (such strategies would be framed within the domains of social support and/or expression of emotion). However, relationships between students and their clinical educators and nurses and medical staff have been widely reported in the literature [12, 27, 31, 32], as being difficult relationships based on a lack of emotional or social support. Both teachers and mentors should be responsible for the proper implementation of coping strategies as basic tools in the skills to be acquired during their competencies in the clinic. The university faculty should not only be aware of the stress levels of students, but also consider how they manage this stress, i.e. whether they use appropriate and effective tools for coping with the same, as this will be key in their development as a nurse. Getting to know the level of stress and/or anxiety that is experienced by our students is important for determining which negative effects should be changed in their behaviors to improve coping.

### Implications for education

Our findings are in line with previous research, highlighting that the study of coping strategies appears instrumental for the prevention of stress. Moreover, it is essential for training in these strategies to begin within the university facilities. These programs could help prepare nursing students to cope with the challenges they are about to face during their clinical rotations. For example, in Spain, none of the universities have a nurse student peer mentoring or support program. This type of peer mentoring program, in which third year students mentor first year students, was implemented in other foreign universities in order to reduce the anxiety experienced by first year nursing students and to facilitate a smooth transition to clinical practice situations [33]. Other strategies have demonstrated to be effective in the management of stress and anxiety in nursing students, such as the use of biofeedback and mindfulness and meditation interventions [34], or emotional freedom techniques [35].

These findings should be considered by nurse educators to create a planning strategy to prevent recurrence of stress based on the use of coping strategies that are more closely correlated with lower levels of perceived stress.

### Limitations

This study has limitations that must be considered when interpreting these results. First, these findings cannot be generalized, as the study was conducted in nursing students of only one faculty of nursing and therefore, the socio-demographic structure of the sample was not necessarily the same as other faculties in Spain. The performance of longitudinal studies conducted over several academic years is recommended as these may reveal changes in perceived stress over time. Further studies based on qualitative techniques would provide more detail regarding the stressor factors and their relationship with levels of anxiety and coping strategies. However, despite these limitations, the results of this study appear to concur with previous findings on this topic.

## Conclusions

In light of these findings, we recommend that the teaching of positive coping strategies should be implemented in the nursing curriculum *prior* to clinical placements. Qualitative research focused on the student’s perception on their clinical experience may be helpful for developing an effective clinical teaching strategy in nursing education.

## Data Availability

The datasets used and/or analyzed during the current study are available from the corresponding author on reasonable request.

## Appendix

**Table 6:** Clinical training progression within the university training program

	1st semester	2nd semester
2nd year	Clinical training I (6ETCs)	Clinical Training II(6ETCs)
3rd year	Clinical training III (6ETCs)	Clinical training IV(6ETCs) Clinical training V (6ETCs)
4 year	Practicum I (25 ETCs)	Practicum II (25 ETCs)

## Abbreviations

ETCS: European Credits Transfer System
PSS: Perceived stress scale
STAI: The State-Trait Anxiety Inventory
CSI: The Coping Strategy Inventory
